# Opportunistic skeletal muscle metrics as prognostic tools in metastatic castration-resistant prostate cancer patients candidates to receive Radium-223

**DOI:** 10.1007/s12149-022-01716-w

**Published:** 2022-01-19

**Authors:** Matteo Bauckneht, Rita Lai, Francesca D’Amico, Alberto Miceli, Maria Isabella Donegani, Cristina Campi, Daniela Schenone, Stefano Raffa, Silvia Chiola, Francesco Lanfranchi, Sara Elena Rebuzzi, Elisa Zanardi, Malvina Cremante, Cecilia Marini, Giuseppe Fornarini, Silvia Morbelli, Michele Piana, Gianmario Sambuceti

**Affiliations:** 1 Department of Health Sciences (DISSAL), University of Genova, Genova, Italy; 2 Nuclear Medicine, IRCCS Ospedale Policlinico San Martino, Genova, Italy; 3 Department of Mathematics (DIMA), University of Genoa, Genoa, Italy; 4 LISCOMP, Department of Mathematics (DIMA), University of Genoa, Genoa, Italy; 5 Medical Oncology, Ospedale San Paolo, Savona, Italy; 6 Department of Internal Medicine and Medical Specialties (Di.M.I.), University of Genova, Genoa, Italy; 7 Academic Unit of Medical Oncology, IRCCS Ospedale Policlinico San Martino, Genoa, Italy; 8 Medical Oncology Unit 1, IRCCS Ospedale Policlinico San Martino, Genoa, Italy; 9 Bioimaging and Physiology (IBFM), CNR Institute of Molecular, Segrate, Milan Italy; 10 CNR-SPIN Genoa, Genoa, Italy

**Keywords:** Metastatic castration-resistant prostate cancer, Skeletal muscle, Sarcopenia, 18F-Fluorodeoxyglucose, Positron emission tomography, Radium-223

## Abstract

**Objective:**

Androgen deprivation therapy alters body composition promoting a significant loss in skeletal muscle (SM) mass through inflammation and oxidative damage. We verified whether SM anthropometric composition and metabolism are associated with unfavourable overall survival (OS) in a retrospective cohort of metastatic castration-resistant prostate cancer (mCRPC) patients submitted to 18F-Fluorodeoxyglucose Positron Emission Tomography/Computed Tomography (FDG PET/CT) imaging before receiving Radium-223.

**Patients and methods:**

Low-dose CT were opportunistically analysed using a cross-sectional approach to calculate SM and adipose tissue areas at the third lumbar vertebra level. Moreover, a 3D computational method was used to extract psoas muscles to evaluate their volume, Hounsfield Units (HU) and FDG retention estimated by the standardized uptake value (SUV). Baseline established clinical, lab and imaging prognosticators were also recorded.

**Results:**

SM area predicted OS at univariate analysis. However, this capability was not additive to the power of mean HU and maximum SUV of psoas muscles volume. These factors were thus combined in the Attenuation Metabolic Index (AMI) whose power was tested in a novel uni- and multivariable model. While Prostate-Specific Antigen (PSA), Alkaline Phosphatase (ALP), Lactate Dehydrogenase and Hemoglobin, Metabolic Tumor Volume, Total Lesion Glycolysis and AMI were associated with long-term OS at the univariate analyses, only PSA, ALP and AMI resulted in independent prognosticator at the multivariate analysis.

**Conclusion:**

The present data suggest that assessing individual 'patients' SM metrics through an opportunistic operator-independent computational analysis of FDG PET/CT imaging provides prognostic insights in mCRPC patients candidates to receive Radium-223.

**Graphical abstract:**

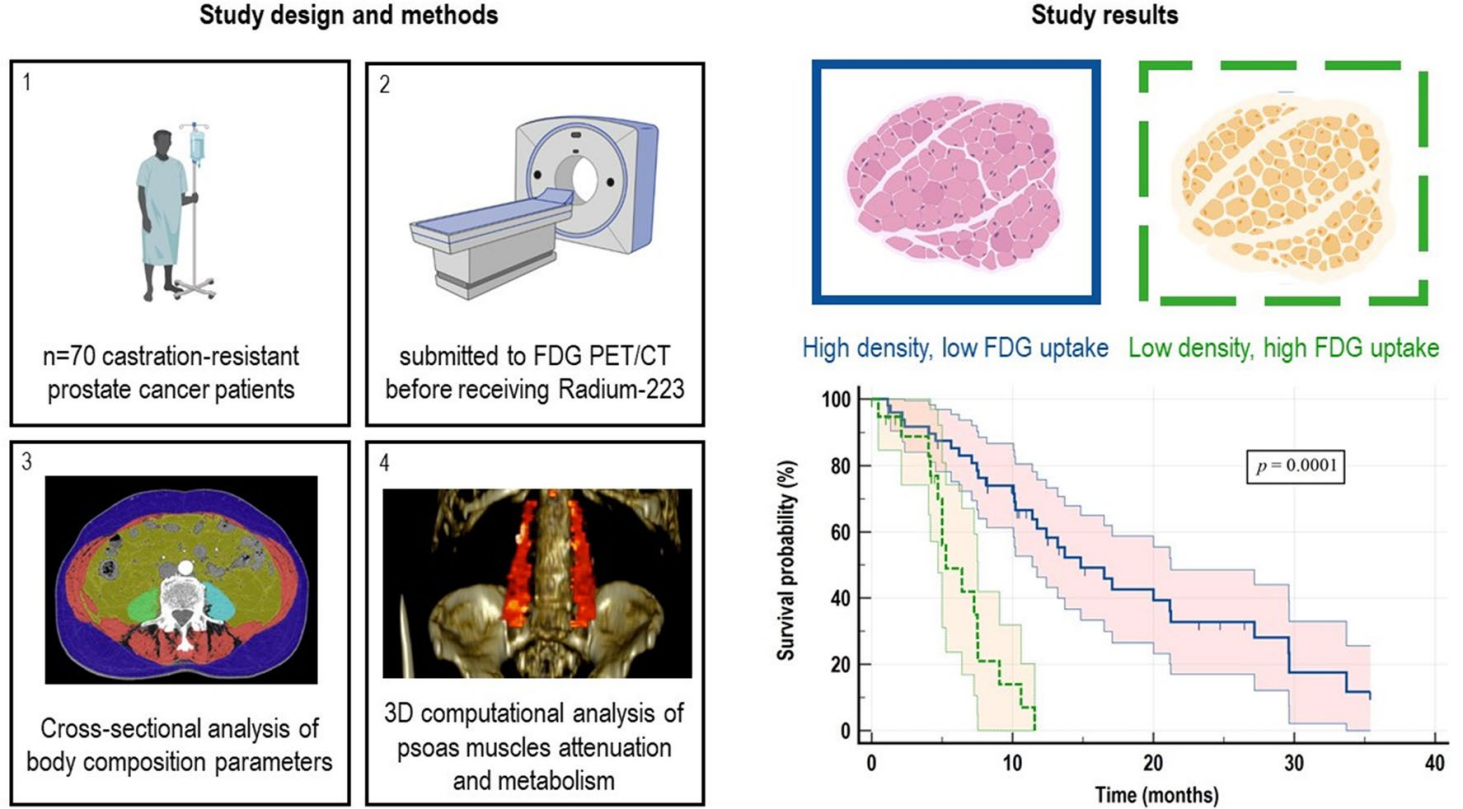

## Introduction

Prostate cancer (PC) represents the most common solid male malignancy in the western world [1]. The clinical behaviour of PC is widely heterogeneous, ranging from hormone-responsive disease to a highly aggressive and treatment-resistant one [1, 2]. This latter setting is often characterized by a metastatic diffusion combined with the loss of hormone sensitivity configuring the clinical scenario of metastatic castration-resistant prostate cancer (mCRPC). In these patients, several treatment options can improve the overall survival (OS) when added to the androgen-deprivation therapy (ADT), including the administration of alpha-emitting radionuclides such as Radium-223 [3–9].

The phase III Alpharadin in Symptomatic Prostate Cancer Patients (ALSYMPCA) trial recruited patients with mCRPC with exclusive bone metastases who received Radium-223 compared to placebo, showing a significantly improved OS in the treated group [10]. Nevertheless, this result was only partially confirmed in clinical practice during the last decade [11–13]. Among the possible explanations of this discrepancy, concerns have been raised about the criteria used for the selection of candidates for this treatment [14]. Therefore, defining prognostic factors able to identify mCRPC patients who will most likely benefit from Radium-223 since baseline, potentially improving this difficult selection process, represents a challenging and crucial issue.

In this scenario, a few studies showed that imaging of ^18^F‐Fluorodeoxyglucose (FDG) uptake with Positron Emission Tomography/Computed Tomography (PET/CT) provides prognostic insights in advanced mCRPC, potentially guiding the systemic treatment selection [15–17]. In particular, the higher is FDG retention in skeletal lesions, the lower are the chances of response to Radium-223 [16, 17]. Nevertheless, PET/CT imaging may opportunistically provide a series of data related to the normal tissues of mCRPC patients, whose analysis might provide prognostic insights independent of the tumour itself.

Among these variables, sarcopenia recently gained attention as a prognosticator in PC [18–21]. Sarcopenia defines a systematic loss of skeletal muscle (SM) mass that decreases below two standard deviations of normal healthy adults [22]. The analysis of the SM area at the level of the third lumbar vertebra (L3) in CT images has already proved to represent an accurate procedure to identify sarcopenia [23]. However, given the multifactorial aetiology underlying muscle wasting, including systemic inflammation [24], FDG PET/CT might represent a potential better descriptor of this muscle disorder.

On these bases, the present study aimed to assess whether SM mass, SM metabolism or their eventual combination, opportunistically derived from FDG PET/CT imaging, are associated with unfavourable OS in a cohort of mCRPC patients’ candidates to Radium-223 therapy.

## Materials and methods

### Study population and design

We performed a retrospective analysis of all consecutive mCRPC patients treated with Radium-223 from January 2015 to November 2021 at IRCCS Ospedale Policlinico San Martino of Genoa, Italy. CRPC was defined as a serum testosterone level of < 50 ng/dl following pharmaceutical castration. All recruited patients were submitted to FDG PET/CT before receiving Radium-223 for prognostic purposes, as indicated by the national guidelines [25]. The study was performed according to the Declaration of Helsinki, Good Clinical Practice, and local ethical regulations. The local ethical committee of Regione Liguria approved the study (Regional Ethical Committee of Liguria—registration number 590/2020). All patients enrolled in the study signed a written informed consent at the time of FDG PET/CT and at the time of each Radium-223 administration, encompassing the use of anonymized data for retrospective research purposes.

### Imaging and treatment procedures

FDG PET/CT was performed according to the European Association of Nuclear Medicine (EANM) Guidelines [26]. Briefly, after a minimum 6-h fasting, a dose of 4.8–5.2 MBq of FDG per kilogram of body weight was injected through a peripheral vein catheter. Patients were placed in a quiet room and instructed to remain still. Data acquisition started ≥ 60 min after tracer injection. Patients underwent low-dose CT from the skull base to the thighs for attenuation-correction and anatomic localization of the FDG-avid lesions, followed by PET imaging. PET/CT studies were performed with two different PET/CT systems (Hirez-Biograph 16; Siemens Medical Solutions, Munich, Germany and Biograph mCT Flow; Siemens Medical Solutions, Munich, Germany). Standard parameters used were CT: 80 mA, 120 kV without contrast; 2.5 min per bed-PET-step of 15 cm; the reconstruction was performed in a 128 × 128 matrix and with a 60 cm field-of-view. PET images reconstruction was obtained using ordered subset expectation maximization (OSEM) algorithms, and attenuation correction was performed using the CT raw data. The entire CT dataset was fused with the 3-dimensional PET images using an integrated software interface (Syngo Image Fusion; Siemens Erlangen, Germany) to create anatomical images superimposed with FDG uptake.

Radium-223 (55 KBq/kg) was intravenously administered every four weeks and continued until disease progression, unacceptable toxicity, death, or patient choice up to six cycles. According to the current EANM guidelines, chemotherapy, Abiraterone, or Enzalutamide were discontinued before the first Radium-223 administration, while patients continued receiving androgen deprivation therapy [27]. As part of our standard protocol, patients were clinically followed-up until death or patient choice after treatment completion.

### Anthropometric measurements on FDG PET/CT images

Anthropometric measurements were performed on PET/CT images using a cross-sectional and a 3D computational approach.

The cross-sectional areas of subcutaneous fat (HU: − 190 to − 30), visceral fat (HU: − 150 to − 50), and SM (HU: − 29 to 150) from the low-dose CT of PET/CT scan at the level of L3 were calculated using a validated freely available online software (www.CoreSlicer.com) [28]. For SM cross-sectional analysis, two consecutive images were recorded in the L3 plane. The sum of the cross-sectional areas of all SM was calculated, averaged, and divided by the square of the height (termed Skeletal Muscle Index, SMI). The formula used was: SMI = L3 SM cross-sectional area (cm^2^)/height^2^(m^2^) [29]. The occurrence of sarcopenia was defined according to international consensus definitions of an SMI < 55 cm^2^/m^2^ for men [29]. Adipose tissue area was calculated as the sum between visceral and subcutaneous fat areas. Visceral-to-subcutaneous fat ratio (VSR) and visceral fat-to-muscle ratio (VMR) were also calculated.

To improve the accuracy of the anthropometric evaluation, an in-house validated operator-independent 3D computational approach based on the Hough transform was also applied [30, 31]. This method can extract the entire recognizable psoas muscles volume from the low-dose CT images of PET/CT scans instead of a single muscle slice at the level of L3. This approach extracts the Psoas volume from its insertion in L2-L3 to the L5-S1 symphysis plane. Extracted volumes, average Psoas HU (HUmean) and HUmean Standard Deviation (HUmean SD) can be thus extracted from the entire muscles in an operator-independent fashion. The extracted binary mask can also be translated to the corresponding FDG PET data to calculate maximum and mean Standardized Uptake Values (SUV) from the entire Psoas muscles volumes of interest.

### Prognostic clinical and imaging data collection

Baseline established prognosticator included patient's age, Gleason Score (GS), International Society of Urological Pathology (ISUP) grade group at diagnosis, serum Prostate-Specific Antigen (PSA), alkaline phosphatase (ALP) and lactate dehydrogenase (LDH) at diagnosis and at the time of imaging, the number of bone metastases at bone scan, the number of previous lines of systemic treatment, and the eventual prior chemotherapy. The maximum standardized uptake value (SUVmax) of the hottest metastatic lesion was obtained from FDG PET/CT images. A volume of interest was then drawn using an SUV-based automated contouring program with an isocounter threshold based on 40% of the SUVmax [32]. The sum of all metastatic lesions identified the total Metabolic Tumor Volume (MTV). In contrast, the sum of the products between volume and the corresponding SUVmean of each lesion determined the Total Lesion Glycolysis (TLG).

### Statistical analyses

The descriptive analyses were conducted using absolute frequency and percentage for categorical variables and by median and range for quantitative variables. Continuous data are expressed as mean ± SD. The study's primary endpoint was the overall survival (OS), which was defined as the time from FDG PET/CT imaging until death from any cause, censored at last follow-up for patients who were alive. The Kaplan–Meier (KM) method was used to estimate the survival curve of OS [33]. Differences were considered statistically significant when the *p* value (*p*) was < 0.05. Univariate and multivariate analyses were performed, assessing clinical, laboratory and imaging parameters in correlation with OS, using Cox proportional hazard regression model, estimating hazard ratios (HRs) and their 95% confidence interval (CI). Only factors with a *p* < 0.10 at the univariable analysis were evaluated in the multivariable analyses for OS. All statistical analyses were performed using the software IBM‐SPSS release 23 (IBM, Armonk, USA) and MedCalc release 12 (MedCalc Software, Mariakerke, Belgium).

## Results

### Patients' characteristics

**Fig. 1:**
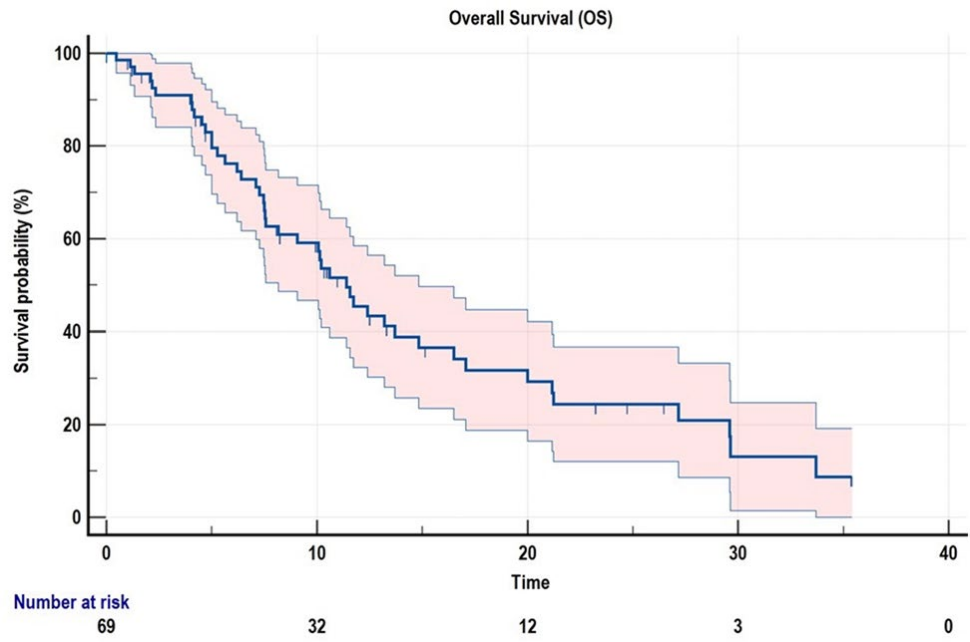
Kaplan–Meier survival function of the study cohort

Seventy mCRPC patients were included in the analysis. Patients' and treatment characteristics are summarised in Table 1. The mean age was 74.81 ± 8.4 years, and patients with ≥ 75 years were 45.7% of the entire cohort. At the time of diagnosis, 35/70 (50%) and 37/70 (53%) had GS ≥ 8 and were metastatic, respectively. Among all patients, 19% received Radium-223 as second-line therapy, while 33.3% and 47.6% received Radium-223 as the third or further line, respectively. Most patients had previously received chemotherapy (58.7%). At the time of data cut-off (November 2021), with a median follow-up of 8.6 months, 46/70 (65.7%) of patients were dead, and the median OS (mOS) was 11.5 months (Fig. 1).

**Table 1:** Patients' characteristics

Clinical characteristics	*n* (%)
Median age, years (range)	74.77 (51.7–89.1)
Median weight, kg (range)	80.00 (54–119)
Median Body Mass Index (BMI)	26.93 (18.5–38.6)
International Society of Urological Pathology (ISUP) grade group	
Gleason Score < 8	23/70 (32.8%)
Gleason Score ≥ 8	35/70 (50%)
Missing	12/70 (17.2%)
Metastases at diagnosis	
No	33/70 (47.2%)
Yes	37/70 (52.8%)
Lab tests at diagnosis	
Prostate Specific Antigen (PSA, ng/mL)	329.87 ± 1561.9
Prior chemotherapy	
No	12/70 (17.2%)
Yes	51/70 (72.8%)
Missing	7/70 (10%)
Lab tests at the time of FDG PET/CT imaging	
Hemoglobin (g/dL)	11.49 ± 1.8
Prostate Specific Antigen (PSA, ng/mL)	391.46 ± 946.7
Alkaline phosphatase (AP, U/L)	234.45 ± 271.72
Lactate dehydrogenase (LDH, U/L)	322.36 ± 322.63
Neutrophil to lymphocyte ratio (NLR)	5.24 ± 4.3
FDG PET/CT parameters	
Standardized Uptake Value (SUVmax) of the hottest bone lesion	8.19 ± 4.2
Metabolic Tumor Volume (MTV, cm^3^)	523.96 ± 591.4
Total Lesion Glycolysis (TLG)	2207.47 ± 2982.8

### Cross-sectional versus computational analysis of SM composition

All 70 mCRPC enrolled patients were classified as radiologically sarcopenic and the mean SMI of the study cohort was 34.27 ± 6.2. Results from univariate and multivariate analyses, including body weight, BMI, cross-sectional and 3D computational body composition parameters, are reported in Table 2. Bodyweight and BMI did not correlate with OS. Among the cross-sectional body composition parameters, SM area significantly correlated with OS, while SMI, adipose tissue area, VSR, and VMR did not reach significance. Among the 3D computational body composition parameters, psoas HUmean and psoas SUVmax resulted prognostic, while psoas volume, psoas HUmean SD and Psoas SUVmean did not predict OS.

**Table 2:** Uni- and multivariable Cox regression analyses including cross-sectional and 3D computational body composition metrics

Variables		Univariate		Multivariate	
		HR (95% CI)	*p* value	HR (95% CI)	*p* value
Clinical data	Body weight (kg)				
	< 80	1.00 (ref)			
	≥ 80	0.76 (0.42–1.37)	0.362		
	Body Mass Index (BMI)				
	< 26.9	1.00 (ref)			
	≥ 26.9	1.41 (0.78–2.53)	0.244		
Cross-sectional data	Skeletal muscle index (SMI, cm^2^/m^2^)				
	< 33.7	1.00 (ref)			
	≥ 33.7	0.64 (0.35–1.16)	0.147		
	SM Area (cm^2^)				
	< 102.8	1.00 (ref)			
	≥ 102.8	0.44 (0.24–0.79)	**0.007**		
	Adipose tissue area				
	< 423.3	1.00 (ref)			
	≥ 423.3	0.82 (0.46–1.47)	0.521		
	Visceral-to-subcutaneous fat ratio (VSR)				
	< 1.32	1.00 (ref)			
	≥ 1.32	1.40 (0.78–2.51)	0.258		
	Visceral fat-to-muscle ratio (VMR)				
	< 2.24	1.00 (ref)			
	≥ 2.24	1.28 (0.71–2.29)	0.406		
3D computational data	Psoas Volume (cm^3^)				
	< 165.4	1.00 (ref)			
	≥ 165.4	0.63 (0.35–1.15)	0.137		
	Psoas HUmean				
	< 29.5	1.00 (ref)		1.00 (ref)	
	≥ 29.5	0.53 (0.29–0.97)	**0.040**	0.44 (0.23–0.82)	**0.010**
	Psoas HUmean Standard Deviation (DS)				
	< 39.6	1.00 (ref)			
	≥ 39.6	1.42 (0.78–2.57)	0.246		
	Psoas SUVmax				
	< 3.9	1.00 (ref)		1.00 (ref)	
	≥ 3.9	2.51 (1.37–4.57)	**0.003**	2.95 (1.57–5.53)	**0.001**
	Psoas SUVmean				
	< 1.1	1.00 (ref)			
	≥ 1.1	1.73 (0.96–3.11)	0.067		

Statistically significant differences were indicated in bold

Specifically, lower SM area, low HUmean and high SUVmax were associated with a poor OS, suggesting that the inferior long-term outcome is predicted by the reduction in SM body content, SM density as well as by the increased SM FDG uptake. The multivariate model including SM area (< 102.8 vs ≥ 102.8 cm^2^), psoas HUmean (< 29.5 vs ≥ 29.5), and psoas SUVmax (< 3.9 vs ≥ 3.9) identified psoas HUmean and SUVmax as independent predictors of longterm OS (both with p ≤ 0.01).

### Association of structural and metabolic SM composition parameters and OS

**Fig. 2:**
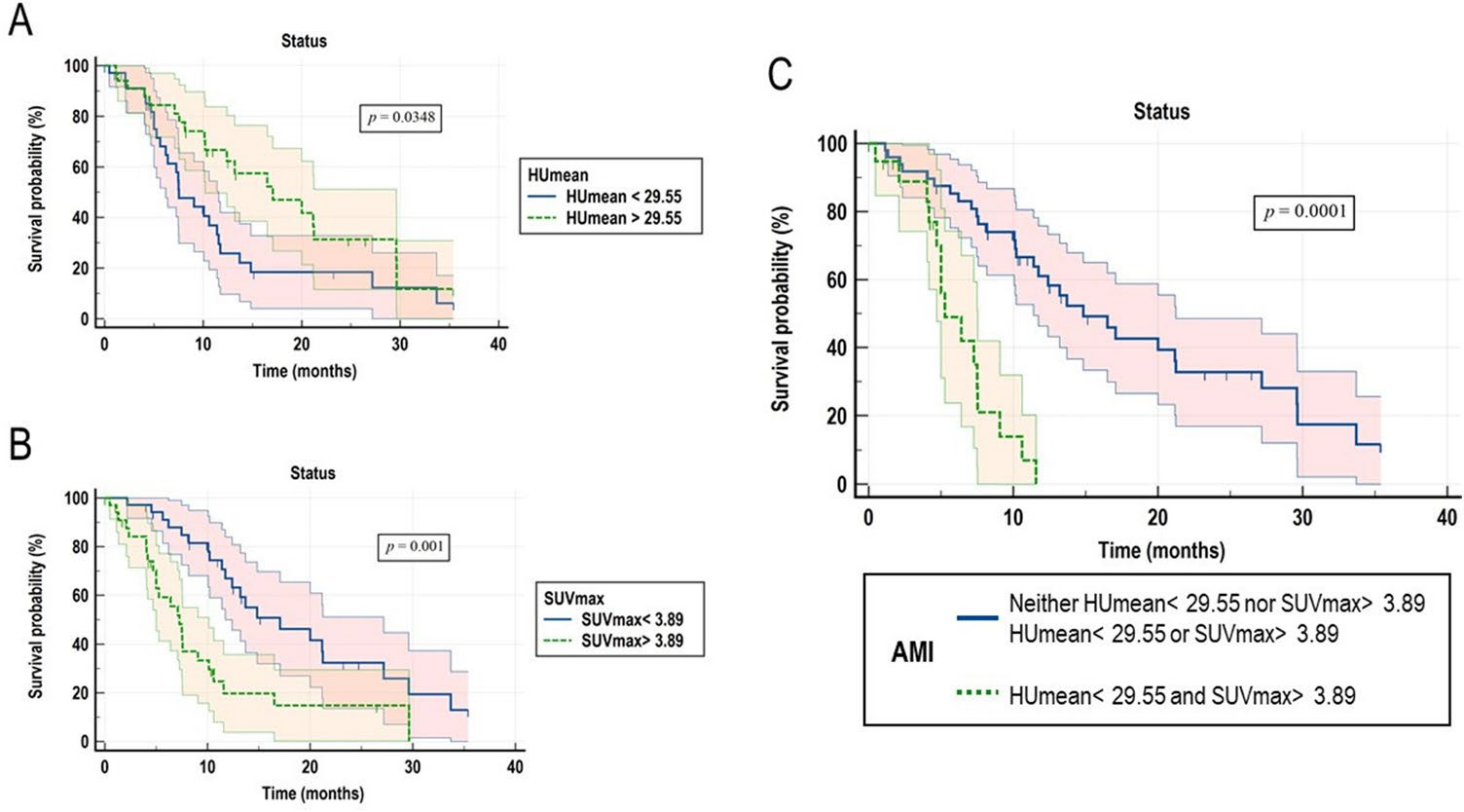
Kaplan–Meier curves for OS according to SM attenuation, metabolic metrics and their combination in the Attenuation Metabolic Index (AMI). Kaplan–Meier curves for overall survival (OS) according to SM HUmean (Panel **A**), SM SUVmax (Panel **B**), and their combination in the Attenuation Metabolic Index (AMI, Panel **C**)

Results from Kaplan–Meier analyses of HUmean and SUVmax, are reported in Fig. 2A, B. The combination of the parameters mentioned above allowed us to identify a novel composite index, which categorized the enrolled cases in two groups with different risks as it follows: low risk (including cases with neither HUmean < 29.55 nor SUVmax > 3.89 and cases with HUmean < 29.55 or SUVmax > 3.89, *n* = 49), and high risk (HUmean < 29.55 and SUVmax > 3.89, *n* = 21). Given both attenuation and metabolic parameters as determinants, the obtained index was named Attenuation Metabolic Index (AMI). Kaplan–Meier curves for AMI are represented in Fig. 2C. Median OS was 16.5 months (95% CI 9.03–23.97 months), and 5.6 months (95% CI 3.81–7.45 months) for the low and the high AMI groups, respectively (*p* < 0.0001). When included in a multivariate model containing acknowledged clinical, lab and imaging prognosticators, AMI resulted in an independent predictor of long-term OS (Table 3).

**Table 3:** Uni- and multivariable Cox regression analyses including clinical, lab and imaging data

Variables	Univariate		Multivariate	
	HR (95% CI)	*p* value	HR (95% CI)	*p* value
PSA at diagnosis				
< 12.34 ng/mL	1.00 (ref)			
≥ 12.34 ng/mL	0.72 (0.39–1.31)	0.289		
International Society of Urological Pathology (ISUP) grade group				
GS < 8	1.00 (ref)			
GS ≥ 8	1.56 (0.82–3.06)	0.189		
Metastases at diagnosis				
No	1.00 (ref)			
Yes	1.03 (0.57–1.86)	0.921		
Previous chemotherapy				
No	1.00 (ref)			
Yes	0.96 (0.53–1.74)	0.906		
Number of previous lines of therapy for CRPC				
1	1.00 (ref)			
2	0.91 (0.01–3.53)			
≥ 3	1.06 (0.01–3.33)	0.963		
PSA at the time of FDG PET/CT				
< 52.64 ng/mL	1.00 (ref)		1.00 (ref)	
≥ 52.64 ng/mL	3.48 (1.79–6.73)	**0.0001**	2.72 (1.25–5.90)	**0.011**
ALP at the time of FDG PET/CT				
< 110 IU/L	1.00 (ref)		1.00 (ref)	
≥ 110 IU/L	5.61 (2.50–12.54)	**0.0001**	2.07 (1.01–4.29)	**0.049**
LDH at the time of FDG PET/CT				
< 229 IU/L	1.00 (ref)			
≥ 229 IU/L	2.46 (1.26–4.783)	**0.008**		
Hemoglobin at the time of FDG PET/CT				
≥ 11.7 g/dL	1.00 (ref)			
< 11.7 g/dL	3.12 (1.64–5.89)	**0.001**		
NLR at the time of FDG PET/CT				
< 3.73	1.00 (ref)			
≥ 3.73	1.39 (0.76–2.54)	0.285		
MTV				
< 250.8 cm^3^	1.00 (ref)			
≥ 250.8 cm^3^	2.51 (1.37–4.61)	**0.003**		
TLG				
< 771	1.00 (ref)			
≥ 771	2.56 (1.37–4.76)	**0.003**		
Attenuation Metabolic Index (AMI)				
Low risk	1.00 (ref)		1.00 (ref)	
High risk	5.19 (2.47–10.09)	**0.0001**	3.12 (1.30–7.47)	**0.010**

Statistically significant differences were indicated in bold

## Discussion

A growing body of literature suggests that the efficacy of Radium-223 is closely dependent on pre-treatment 'risk stratification [14]. Therefore, several studies investigated many potential baseline prognostic factors, whose application might optimize the patient's selection process [34]. Coherently with the existing literature, in the present study we observed a prognostic role of baseline PSA, ALP, LDH, and haemoglobin in a cohort of mCRPC candidates to receive Radium-223 [35–41]. Similarly, in agreement with the existing literature [16, 17], the extent and metabolic activity of the metastatic burden, as described by MTV and TLG derived from FDG PET/CT images, predicted OS. On the other hand, we observed that baseline SM structural and metabolic metrics as well as their combination correlate with long-term survival, regardless of the clinical, lab and imaging descriptors of the tumour extension. These data can be opportunistically obtained from FDG PET/CT images, without additional imaging examination costs or radiation exposure.

In the last years, there has been an increasing interest in assessing opportunistic biomarkers from routine, standard of care imaging. An emblematic example is represented by the imaging-based assessment of sarcopenia, which has been correlated with long-term OS in a wide range of solid cancers [42]. The occurrence of sarcopenia has also been related to the increased risk of postsurgical complications [43] and systemic treatment toxicity [44], leading to the notion that assessing the sarcopenic status before treatment may guide customised strategies and support tailored treatment decision-making. A robust prognostic impact of sarcopenia has also been shown in PC, in which this condition is present in over 60% of patients [21, 45, 46]. However, at the later stages of the disease (i.e., in heavily pre-treated mCRPC), the prevalence of sarcopenia can be considerably increased [21, 45]. Coherently, in the present study, all the enrolled patients met the radiological criteria for sarcopenia. This finding suggests that the use of the conventional radiological definition of sarcopenia may be an inaccurate prognosticator at these stages. On these bases, we extended the conventional anthropometric evaluation of body composition to a vast range of cross-sectional and computational parameters.

From the methodological point of view, this approach allowed us to observe that the 3D computational analysis of SM overcomes the cross-sectional assessment in terms of the prediction of the long-term OS. This difference might be related to the higher reproducibility of volumetric methods compared to cross-sectional area measures, given to the operator-independency of the latter approach. Further, imaging measures from a single axial section may not be representative of measures derived using volumetric analyses, as already documented in other solid tumours [47] as well as in anthropometric evaluations [48]. Finally, the computational analysis allowed us to extract FDG PET/CT-derived parameters from SM volumes.

The observed prognostic power of FDG-based measures of SM is of potential interest from the pathophysiological perspective. Indeed, it has been suggested that the reduction of SM HUmean in mCRPC (reflecting the increase in fat content) may result from the prolonged androgen deprivation, as castrate levels of testosterone may lead to reduced muscle mass and increased subcutaneous and visceral adipose tissue [49]. However, given the observed prognostic value of the increased FDG uptake in the sarcopenic SM, we presume that this phenomenon might also involve the occurrence of SM inflammation. Indeed, several previous studies highlighted the occurrence of a systemic inflamed state in heavily pre-treated mCRPC [11, 17, 39, 50]. On the other hand, a direct causal role of inflammation has been shown in the age-related decline of SM mass [51]. Consistently, a few previous studies correlated SM FDG uptake with clinical conditions possibly mediated through inflammatory mechanisms [52–55]. On the one side, the inflamed SM might be FDG-avid due to the high content in inflammatory infiltrates. Alternatively, given the oxidative environment promoted by SM inflammation [56], the increased FDG uptake may at least partially reflect the activation of NADPH generation by a pentose phosphate pathway selectively located within the endoplasmic reticulum, as previously documented in cancer cells [57, 58], neurons [59], astrocytes [60], cardiomyocytes [61], and, more importantly, in the SM [62, 63].

The present study has some limitations. First, as in any retrospective study we cannot a priori exclude the eventual occurrence of selection biases. However, we enrolled a naturalistic group of mCRPC patients' candidates to receive Radium-223 aiming to reflect the real-world clinical practice. This choice allowed us to identify a cohort of mCRPC with a remarkably high prevalence of sarcopenia. This result might be related to the late stage of the disease of patients receiving Radium-223 in the clinical practice, which is at least partially related to the restriction use promoted by the European Medical Agency (EMA) in 2018 [64]. However, we cannot assume the same prevalence of sarcopenia as generalizable to a broader group of mCRPC patients. Similarly, given the retrospective enrolment we are unable to estimate the exact duration of ADT prior to initiation of Radium-223 in enrolled patients. Owing to the documented correlation between ADT duration and SM loss [49] further studies are warranted to estimate the impact of this variable on obtained results. As a final consideration, CT-derived SM metrics were obtained from the low-dose CT of FDG PET/CT studies instead of using high-resolution CT. However, given the results of a recent study by Albano et al. [65], showing a strong correlation between SM metrics derived from low- and high-dose CT, we assume that this choice might have had a low impact on the reproducibility of our results.

## Conclusion

The present data suggest that assessing individual patients SM metrics through an operator-independent computational analysis of FDG PET/CT data may potentially guide customised strategies and support tailored treatment decision-making in mCRPC patients candidates to receive Radium-223.

## Data Availability

The data that support the findings of this study are available from the corresponding author upon reasonable request.
